# Transactions of the Chicago Medical Society

**Published:** 1885-11

**Authors:** 


CHICAGO MEDICAL SOCIETY.

*Stated Meeting, Oct, 5, 1885*, the President, C. T. PARKES, M. D., in the chair.

### OFFICIAL REPORT.

*Intubation of the Larynx, with a Report of Five Cases*, was the title of a paper read by Dr. F. E. WAXHAM. The paper was supplemental to one read before the Society on April 20th, in which the operation was minutely described. Dr. WAXHAM exhibited a larynx with the tube in situ. He described the manner of performing the operation, as follows, by the nurse holding the child in her lap, with the hands at the side, an assistant firmly holding the head backward. The mouth is held open by a gag placed on the left side between the teeth. The tube, armed with a silk bridle, well waxed, is now secured to the introducing instrument. The right hand manipulates the instrument, while the index finger of the left hand guides safely and quickly the tube over the epiglottis into the larynx, when the introducing instrument is removed and the tip of the finger presses the tube well down into the larynx. We make sure the tube is in proper position by the easier breathing, the tube remaining stationary, and by coughing on the patient attempting to swallow water. The bridle of silk is apt to produce violent coughing, and is generally removed. The latest improvements in the tubes consist in an enlargement of the head of the tube with a backward curve, preventing the tube from slipping into the trachea and allowing the epiglottis to fall during the act of deglutition. There is also an enlargement in the center of the tube, allowing it to be more easily extracted. Dr. WAXHAM reported in detail five cases of croup treated by intubation. One case recovered, one died six days after intubation from pneumonia; the result of unfavorable surroundings. The other cases were not such that recovery could be definitely expected. Dr. WAXHAM then presented the history of the five cases in detail, after which Dr. H. T. BYFORD opened the discussion by saying he had the pleasure of seeing the case reported in which there was a complete recovery. In the contrast between this operation and tracheotomy there are many points in favor of intubation, and there are not many cases in which tracheotomy is indicated that intubation is not, one of its chief advantages being its simplicity. The first case of tracheotomy he had ever performed was a success and gave him a great deal of encouragement, but the next was such a terrible case, and a failure, that he was discouraged. He had assisted at several tracheotomies, but the difficulties of the operation, the trouble of overcoming the prejudice of the parents against the operation, and the difficulties and bad results following, had caused him to *abandon the operation as of little use except in good cases. But when he saw this case, with all the absence of numerous attendants and paraphernalia* in the after-treatment, and the comfort and freedom of the patient, he was greatly astonished. The simplicity and safety of the operation and the comfort afterwards, the fact that the consent of the parents can be easily and early obtained, that failure to relieve will not bring discredit upon the physician, and that the tube opens in the throat instead of the external air, leaves no doubt in his mind that intubation, whenever it can be successfully accomplished, will supersede tracheotomy in private practice.

Dr. W. E. CASSELBERRY said he was in constant attendance on one of the cases which terminated unfavorably, but the effect from the operation was such as to convince him of its utility in many cases. In this case the former physicians in attendance had thought the patient had recovered from diphtheria, but the membrane later invaded the trachea. The young child was *in extremis mortis* and it was decided tracheotomy would be of no avail, and it was not thought intubation would be much better, but in order to give the child a chance it was done. The child lived twenty-four hours, and its last hours were comparatively comfortable. In this case there was considerable difficulty in the introduction of the tube, and it was a lesson to him that practice in introducing the tube on the cadaver might obviate many difficulties in introducing it on the living subject. In the case of this young child the tube attached to the instrument for introducing it made too short an angle to be easily introduced. The idea suggested itself to have a joint in the introducing instrument so as to be able to easily pass the curve of the pharynx. The tube caused no cough or difficulty in swallowing, and was easily withdrawn. A German physician lately states that in 111 cases of tracheotomy under his control, 63 recovered, and an American physician notes 20 cases, of which 9 recovered. It seems from these statistics that tracheotomy is not to be discarded, but we nevertheless will find a large field for intubation of the larynx Intubation will be preferable in young children not apt to recover from tracheotomy, in diphtheritic cases, and in cases when the friends object to tracheotomy.

Dr. R. G. BOGUE said: I have happened to have something to do with tracheotomy. While there are a great many inconveniences attending the operation and the care of the patients, subsequently, there certainly has been a good deal to commend in its performance in many cases. The number of recoveries after tracheotomy are not few. The gentleman preceding me referred to statistics showing a larger percentage of recoveries than I had happened to know. But those who have operated a goodly number of times have good reason, from its success, to resort to it in many cases. Intubation is a simple operation compared with tracheotomy, and will recommend itself in many ways, and if it proves to be of equal success in saving life, it should be used in preference to tracheotomy. Many reasons arise why it should be used. It is not a formidable operation; parent's consent to it can be easily obtained, and the relief obtained by intubation seems as great as in tracheotomy. After each tracheotomy there is a period of rest and quiet and apparent promise of success, for a period of twenty-four to thirty-six hours, then an extension of the disease into the deeper air passages, or some complication destroys the life of the patient. The benefit of intubation with only this alleviation is apparent. It is to be hoped after a more extended trial it will prove to be of as much, if not more, service than tracheotomy, and it will commend itself to the profession.

Dr. G. C. PAOLI said Diffenbach, of Berlin, was the first to use intubation in diphtheria and croup, and a Parisian physician tried it at the time, each without the knowledge of the other's experiments. Diffenbach used an india-rubber tube, but he as well as the Parisian physician abandoned intubation. This is a different method, it is true, but it can never be recommended until we have statistics from those having great experience, in hospitals especially, to prove it preferable to tracheotomy.

Dr. D. W. GRAHAM commended the report as being an effort in the right direction. It shows that intubation has some merit, as a means of treating obstruction of the larynx, and that it is destined to become at least a partial substitute for tracheotomy in diphtheritic croup. From a theoretical standpoint it would seem that there would be some liability of these tubes causing œdema of the larynx, if retained in place any length of time, on account of the mechanical pressure on the veins of the mucous membrane. Future observation will show whether they are entirely harmless. However, there does not appear to have been any trouble in this respect in the cases reported.

If this method should become established and recognized, as it now promises, it would and ought to be counted as a new procedure, notwithstanding what Dr. PAOLI has said about the efforts of the older surgeons to put the same idea into practice, for whatever has been attempted heretofore in this direction has proved fruitless.

Dr. WAXHAM, in closing the discussion, in answer to various questions, said the longest time the tube was worn continuously was six days. Dr. O'DWYER reports two cases, terminating favorably, in which the tubes had been worn ten days. He never found any œdema of the larynx caused by the wearing of the tube. In very young children it is necessary to remove and cleanse the tube. Older children, if not exhausted by disease, will expectorate freely. The previous attempts at intubation in France were not successful, but they were not according to the methods now employed. TROUSSEAU discouraged intubation, and thus the French physicians were influenced against it. The tubes must be thin, but their weight is unimportant. He had never found it necessary to use cocaine in introducing the tube, as this operation is generally easily and quickly done.

*Laparomoty in a Case of Gun-shot Wound of the Intestines* was the title of a paper read by Dr. AUGUSTUS V. PARK. He said: M. S., a butcher-boy, aged 16, of slight stature, formerly in poor but lately in good health, was shot on September 1st, 1885, at 3:30 P. M. A pistol ball, of calibre 22, fired from a distance of forty-five feet, entered the abdomen at a point midway between the symphysis pubis and umbilicus, two inches to the left of the median line. The patient was removed in a farmer's spring wagon from the place where he was shot to his home, a distance of seven miles. A dressing was applied, and at 1 P. M., the next day, he was taken to the Michael Reese Hospital. The patient arrived nearly exhausted; his temperature was 100° F., pulse 130, weak and intermitting. His respirations were 30, his abdomen tympanitic especially high on left side. There was no liver dullness, giving rise to a theory that the liver was crowded upward by extravasated blood. At 1:30 P. M. laparotomy was performed, the incision being made directly over the seat of the wound. We could not find any wound of the peritoneum, or where the ball passed through it. As the peritoneum was opened, decomposed blood rushed through the opening with great force. Blood and blood-clots which quickly formed were removed with sponges; the intestines were drawn out and examined for wounds. The first wound found was an abrasion, the ball not having entered the intestine. There was but little hemorrhage, and the wound was closed by the interrupted cat-gut suture. The second wound, half inch in diameter, opened directly into the intestine. A small mesenteric artery was found divided and tied. All hemorrhage ceased. The wound was closed by interrupted suture; no further injury could be detected. The abdominal cavity was cleansed with one per cent. solution of carbolic acid; the intestines were washed, carefully examined and returned. The abdominal incision was closed by two sets of sutures, the peritoneal surfaces were approximated and closed by continuous suture.

At 5:30 on the morning after the operation the patient died. Seven hours later an autopsy revealed commencing peritonitis, the small intestines being apparently agglutinated together. A few blood-clots and a quantity of extravasated blood were found in the peritoneal cavity on the left side. A contused wound of the rectum was found near the sigmoid flexure, the ball being deflected from this position into the muscular tissue below, where it was found imbedded. This case justifies the opinion of various eminent surgeons that we cannot tell the direction the bullet takes from the position of the wound of entrance, or exit. From the conditions existing in this case, he was of the opinion the case would have terminated favorably had he been able to perform, with antiseptic precautions, laparotomy immediately after the injury.

Dr. F. E. WAXHAM said Dr. PARK was entitled to a great deal of credit for presenting to the Society his paper and the specimen, because it is the report of a case which terminated unfavorably. He thought the chances of the patient would have been better if he had been allowed more quiet. His frequent removals must have loosened the blood clots and increased the hemorrhage and prolonged the shock. If he had recovered he would have thought it almost miraculous, for it is one of the maxims of abdominal surgery to have complete and perfect quietude for the patient.

Dr. R. TILLEY said the study of gun-shot wounds of the abdomen is interesting to every member of the profession, no matter in what particular direction his favorite studies may lead. Any one of us may find ourselves confronted with the responsibility associated with such cases when delay in action may be culpable. Relative to the case before us, he should not only not consider a recovery miraculous, but deem the conditions associated with it more favorable than, on the average, can be expected. One of the conclusions formulated by our President before the American Medical Association in Washington in 1884 is, in opening the abdomen to look for gun-shot wounds, the incision should be in the median line, regardless of the bullet wound. This procedure certainly facilitates efficient inspection, but in the present case it was ignored. He regretted that the cause of failure of the operation has not been thrown into stronger relief, and he felt like asking our President, Dr. C. T. PARKES, to formulate the lessons he would draw from the failure of this operation. Of course, the case will go on record as one of operation after gun-shot wound of the abdomen associated with failure, and will tend to develop hesitation in the mind of the general practitioner about a class of cases which, in his opinion, called for urgent, prompt operating.

Dr. BOGUE said there were a few lessons to be learned from this case. One is the advantage which would follow an early operation, before the blood, or fluid in the abdominal cavity, decomposes. An operation should be made before the irritation from this source is severe. Another lesson is the necessity of a thorough exploration of the abdominal cavity for the purpose of discovering and removing any foreign substance which may be in it. It is necessary to control hemorrhage, by opening the abdomen and having free access to every part of it.

Dr. J. H. ETHERIDGE said that it will be noticed the pulse-rate was high after the operation, which lasted two hours. The question arises, if we cannot account for death on the opinion that it was due to the action of the ether on the cardiac nervous system. Was there acute poisoning from ether? Or was death caused by septicæmia? He wished to thank Dr. PARK for the report of this case, because it is from the reporting of these unsuccessful cases we obtain the most benefit. He did not believe it would deter any one from doing abdominal surgery, as it had already taken such a rank that the report of one unsuccessful case would not intimidate any surgeon, but enable him to steer clear of difficulties others may have encountered.

The President said: Your chairman feels somewhat diffident about making any remarks, because his experience in connection with gun-shot wounds of the abdomen was solely in connection with the results of experiments upon the lower animals.

There is one fact demonstrated by this case, and it stands out in all the cases, of which I know, operated upon in man, which corroborates the results of the experiments made by myself, and that is, the necessity of free incision through the median line of the abdomen, without any reference to the course of the bullet, as the best way to get at the injury so as to determine its extent, and to apply the means of repair as well as to secure a clean abdomen. Another item mentioned in the case is the one that blood flowed freely from the bullet wound while the patient was in the erect position and ceased when he was recumbent. As the bullet passed through parts of little vascularity, this item points to the wounding of some large vessel internally (as was found), and becomes a point of value in the question of perforation. This question of perforation is no easy one to settle positively, even in the best of hands. I am inclined to agree with Dr. WAXHAM in the opinion that it was not the best plan to remove the patient from his home before operating, notwithstanding his bad surroundings. We must take into consideration the fact that his patient was accustomed to his surroundings, and far less likely to be harmfully affected by them than by the danger incidental to the jolting movements of removal.

Some of the accidents of the case I am sure would have been avoided by obeying the rule of open incision in the median line. Post mortem showed considerable old blood in the cavity; this would have been found and removed. The paper states no extravasation of bowel contents was noticed; the non-existence of such condition has doubt thrown upon it by the condition found in post mortem. The wound in the rectum would have been discovered, and the action of the bowel displayed shows an untouched perforation of its walls; probably the wound of entrance of the bullet. The exit wound is sewed up.

The manner of closing the external incision as well as the bowel wounds should be such as to *save time* in the operation, by using the continuous cat-gut suture for small bowel wounds and single through and through suture of the abdominal incision. It is pure waste of time to unite the latter in layers.

It is a matter of some pride and great pleasure to me to know that the principles enunciated by me as the results of experiments on the lower animals (especially as they are ridiculed by some) have so recently been put to a severe but successful application upon the human body. Dr. BULL, of New York, had a successful case of nine perforations, and Dr. J. B. HAMILTON, of Washington, D. C., also a successful case with eleven perforations.

In Dr. HAMILTON'S case, the only bad happening arose from the formation of a blood tumor—probably, as Dr. HAMILTON says, forming from a grazed surface, the bleeding from which could not be controlled. This was subsequently opened through the rectum and the patient recovered. But it is interesting to notice that the patient was in greater danger of his life from this mass of blood than from the wounds in the intestine after they had been closed. It shows also how necessary it is to prevent bleeding by securing, if possible, all bleeding points.

After the pathological specimen was examined the Society adjourned.

---

TRANSACTIONS OF THE CHICAGO GYNÆCOLOGICAL SOCIETY.

